# Uterine Endometrium Microbiome in Women with Repeated Implantation Failure Complicated by Endometriosis

**DOI:** 10.3390/jcm13164605

**Published:** 2024-08-07

**Authors:** Yosuke Ono, Yuta Kobayashi, Shigeki Shimada, Yoshiyuki Fukushi, Osamu Yoshino, Shinichiro Wada, Hideto Yamada

**Affiliations:** 1 Department of Obstetrics and Gynecology, University of Yamanashi, 1110 Shimokawahigashi, Chuo 409-3898, Yamanashi, Japan; nadal.babolat@hotmail.co.jp (Y.O.); oyoshino624@gmail.com (O.Y.); 2 Department of Obstetrics and Gynecology, Teine Keijinkai Hospital, 1-40, 12-chome, Maeda, Teine-ku, Sapporo 006-8555, Hokkaido, Japan; kobayashi-yu@keijinkai.or.jp (Y.K.); kohta294@yahoo.co.jp (Y.F.); wa_shin_2002@yahoo.co.jp (S.W.); 3 Department of Obstetrics and Gynecology, Mommy’s Clinic Chitose, 2-1-13 Shinano, Chitose 066-0038, Hokkaido, Japan; shimashige@hotmail.co.jp; 4 Center for Recurrent Pregnancy Loss, Teine Keijinkai Hospital, 1-40, 12-chome, Maeda, Teine-ku, Sapporo 006-8555, Hokkaido, Japan

**Keywords:** *Dialister*, endometriosis, endometritis, microbiome, repeated implantation failure, *Streptococcus*, 16S ribosomal RNA

## Abstract

**Objectives**: This prospective study evaluated whether endometriosis is associated with chronic endometritis (CE) and affects the uterine endometrium microbiome (UEM) in women with repeated implantation failure (RIF). **Methods**: Forty-three women with RIF were divided into 12 with endometriosis (EM) and 31 without endometriosis (non-EM). The UEM was examined by 16S ribosomal RNA (rRNA) sequencing, and CE was determined by CD 138 staining (plasma cells > 5.15/10 mm^2^) simultaneously. **Results**: The EM group had a higher bacterial number (EM vs. non-EM; median [range], 6.5 vs. 3 [3–11, 1–16], *p* = 0.009), while the frequency of *Lactobacillus* species did not change. The rates of presence of *Dialister* (41.7% [5/12] vs. 3.3% [1/31], *p* = 0.004) and *Streptococcus* species (58.3% [7/12] vs. 16.1% [5/31], *p* = 0.017) were higher in the EM group. The prevalence of CE did not differ between the two groups. Multivariable logistic regression analysis revealed that the presence of *Dialister* species (odds ratio, 10.97, 95% confidence interval, 1.17–249.37, *p* = 0.036) was associated with endometriosis. In the EM group, five women with *Dialister* species had a higher number of bacterial species (10 vs. 5 [6–11, 3–7], *p* = 0.021) and higher Shannon diversity index (0.50 vs. 0.20 [0.19–1.39, 0.03–0.46], *p* = 0.026) than seven without *Dialister* species. **Conclusions**: *Dialister* and *Streptococcus* species, and the increased number of bacterial species in UEM may be related to the pathogenesis of RIF complicated by endometriosis.

## 1. Introduction

Endometriosis, which affects 10–15% of women of reproductive age, is a lifelong condition that reduces a woman’s quality of life and causes dysmenorrhea, pelvic pain, infertility, and ovarian cancer, with an increased risk of future cardiovascular events [1,2,3,4]. Endometriosis is an estrogen-dependent pelvic inflammatory disease thought to affect gynecological organs and is a cause of infertility; however, the complex mechanism and variety of factors involved limit our understanding of the condition. Endometriosis is considered a chronic inflammatory disease, and immune abnormalities in its pathogenesis have been pointed out [5]. Although the gut microbiome in women with endometriosis is associated with pathogenesis, immune dysfunction, and inflammatory autoimmune diseases [6,7], the intrauterine microbiome has not been evaluated extensively because of the limited detection capability of bacterial culture methods.

Recently, microbiome analyses with 16S ribosomal RNA (rRNA) sequencing using a next-generation sequencer have been performed in the reproduction field and have made progress in the study of uterine endometrium microbiome [8,9]. Chen et al. examined the microbiome of the uterine cervix, uterine lumen, fallopian tubes, and peritoneal fluid along the female reproductive tract using 16S rRNA sequencing analysis and demonstrated that the microbial community is present in each area [10]. Franasiak JM et al. first examined the microbiome of post-embryo transfer catheter tips in ART patients by 16SrRNA sequencing analysis and found that the most common bacteria were *Lactobacillus* species [11]. Moreno et al. first showed that there was a correlation between the endometrium microbiome and subsequent reproductive outcome. They reported that rates of implantation, pregnancy, and live births after in vitro fertilization embryo transfer were higher in women with *Lactobacillus*-dominant microbiome (>90%) in the endometrium than in those with non-*Lactobacillus*-dominant microbiome, using 16S rRNA sequencing [12]. Liu Y et compared the uterine endometrial microbiome in women with chronic endometritis (CE) associated with implantation failure to uterine endometrial microbiome in women without CE and demonstrated that the percentage of *Lactobacillus* species was significantly decreased in women with CE than that in non-CE (CE vs. non-CE, 1.89% vs. 80.7%) [13]. Recent studies also suggest that abnormalities in the uterine endometrium microbiome are associated with implantation failure; therefore, evaluation of the endometrium microbiome is considered to be important for improving the reproductive outcomes in infertility [14,15,16]. We also examined the uterine endometrium microbiome and CE in women with repeated implantation failure (RIF), recurrent pregnancy loss (RPL) and fertile controls, and revealed increases in the prevalence of CE in women with RPL and in percentages of *Lactobacillus iners* and *Ureaplasma* species in women with CE [17].

The generally accepted theory of endometriosis is that the endometrium refluxed to the pelvic cavity via the fallopian tubes during menstruation grows, proliferates, and develops, and the intraperitoneal cavity of women with endometriosis has traditionally been thought to be sterile. However, a technique using 16S rRNA sequencing identified bacteria in the intraperitoneal fluid and menstrual blood of women with endometriosis [18]. This suggests that the bacteria originate in the uterine cavity and that uterine endometrium microbiome may be involved in the development of endometriosis. Since an increased number of bacterial species at various sites is associated with endometriosis, the endometrial microbiome may be related to endometriosis development [19].

However, studies on the uterine endometrium microbiome in women with endometriosis are still limited, particularly for women with infertility [10]. RIF is an intractable form of infertility believed to be caused by various factors, such as a fertilized egg, uterus, immunity, hormones, and other unknown factors [20].

The intrauterine environment is important during the implantation of a fertilized egg into the endometrium; therefore, the intrauterine microbiome may be involved in the pathophysiology of implantation. In this study, we hypothesized that endometriosis is involved in the pathogenesis of CE and affects the uterine endometrium microbiome in women with RIF. To evaluate this, we compared the uterine endometrium microbiome and the prevalence of CE between women with RIF complicated by endometriosis and those without endometriosis.

## 2. Materials and Methods 

### 2.1. Study Participants

This prospective study was conducted between March 2021 and December 2023 according to the guidelines of the Declaration of Helsinki, and was approved by the Institutional Review Board of Teine Keijinkai Hospital (No. 2-020387-00, date of approval: 14 March 2021), and we obtained informed consent from all participants. We defined RIF as the failure to achieve pregnancy after ≥2 embryo transfers, and 43 women with RIF were enrolled in this study. Of these 43 women, 12 had endometriosis (EM group), whereas 31 had no endometriosis (non-EM group). None of the women with RIF had received prebiotics/probiotics within 3 months of study participation. Clinical information was obtained from electronic medical records. Of the 12 women with endometriosis, 6 had ovarian endometrioma, 2 had adenomyosis, 2 had pelvic endometriosis, 1 had ovarian endometrioma and adenomyosis, and 1 had adenomyosis and pelvic endometriosis (Table 1). Endometriosis was diagnosed based on magnetic resonance imaging, intraoperative findings, and/or postoperative pathology.

### 2.2. Study Protocol and Procedures

Uterine endometrium specimens were obtained via aspiration during the mid-luteal phase, confirmed by transvaginal ultrasonography and the last menstrual cycle, as previously reported [17]. The samples were placed in the middle part of the aspiration tube and soaked in an OMNIgene^®^-VAGINAL microbiome kit (DNA Genotek Inc., Ottawa, ON, Canada). They were promptly sent to Varinos Inc. (Tokyo, Japan), where the uterine endometrium microbiome was analyzed using 16S rRNA sequencing.

The 16S rRNA gene is a component of the small subunit of the ribosome, which is common to bacteria and archaea. The gene has a conserved region, which is common to all bacteria, and a highly mutated region; 16S rRNA sequencing reveals the structure and diversity of microbial communities by analyzing specific regions of the gene. Conserved regions and variable region 4 (V4) of 16S rRNA, were amplified by polymerase chain reaction (PCR) using DNA extracted from tissue specimens [21]. The amplified PCR samples were identified following the Illumina 16S Metagenomic Sequencing Library Preparation protocol [22].

For histopathological findings of CE, after defining CD138-positive cells in the endometrium as plasma cells by immunohistochemical staining and counting the number of CD138-positive cells/10 mm^2^, CE was diagnosed when the plasma cell count was >5.15/10 mm^2^, according to the criteria set by Liu [13], who was the first to demonstrate an association between CE and uterine endometrium microbiome in women with infertility. Histopathological analyses for CE were performed at Sapporo Clinical Laboratory Inc. (Sapporo, Japan). Endometriosis was diagnosed by magnetic resonance imaging examination when the lesion appeared as a single or multifocal cystic lesion with a high signal on both T1- and T2-weighted images or as a uniform high signal on a T1-weighted image and a low signal, called shading, on a T2-weighted image, and was not suppressed on fat-suppression imaging.

### 2.3. Biodiversity of Bacterial Community and Shannon Diversity Index

The biodiversity of a bacterial community in a certain niche can be characterized and quantified by its richness and evenness [23]. Species richness denotes the number of bacterial species present, while evenness pertains to the relative abundance of these organisms. The relative abundance of bacterial species reflects their relative dominance, and mathematical parameters such as species richness and evenness serve as comparative measures. The Shannon diversity index, a widely employed estimator of species richness and evenness [24], was utilized to compare the diversity of the uterine endometrium microbiome.

### 2.4. Statistical Analysis

Data were analyzed using JMP version 16 (SAS Institute Inc., Cary, NC, USA). Categorical variables were compared by Fisher’s exact test and the Mann–Whitney U test. Patient backgrounds, the number of plasma cells/10 mm^2^ with CD138 staining, the frequency of CE (Liu’s method), and the uterine endometrium microbiome were compared between EM and non-EM groups. Multivariable logistic regression analysis was performed to determine the bacterial species associated with endometriosis. All *p*-values were two-sided, and *p*-values < 0.05 were considered significant.

## 3. Results

### 3.1. Chronic Endometritis and Uterine Endometrium Microbiome in RIF with Endometriosis

The clinical characteristics and backgrounds of EM and non-EM groups were compared (Table 1). No differences in age, body mass index, gravidity, parity, number of previous miscarriages, and number of implantation failures were found between the two groups.

The frequency of CE and the uterine endometrium microbiome were compared between EM and non-EM groups (Table 2). The prevalence of CE determined by Liu’s method (plasma cell count > 5.15/10 mm^2^) or the number of CD138-positive cells per 10 mm^2^ was not different between the two groups. The EM group had a higher number of bacterial species than the non-EM group (median [range] 6.0 vs. 3.0 [3–11, 1–16], *p* = 0.009); however, there was no significant difference in the Shannon diversity index. There were no significant differences in the relative dominance rate of *Lactobacillus* species or in the frequency of the *Lactobacillus*-dominant microbiome defined as >90% of the relative dominance rate of *Lactobacillus* species. The relative dominance rate of *Lactobacillus crispatus* was lower in the EM group (median 0 [range 0–98.2]) than in non-EM group (0.15 [0–99.9]); however, the difference was not significant (*p* = 0.053). Among the infertility-associated bacteria, the rate of presence of *Dialister* species (41.7% [5/12] vs. 3.3% [1/31], *p* = 0.004) and *Streptococcus* species (58.3% [7/12] vs. 16.1% [5/31], *p* = 0.017) in the EM group was higher than in the non-EM group. The rates of presence of *Ureaplasma*, *Mycoplasma*, *Gardnerella*, *Prevotella*, *Atopobium*, *Bifidobacterium*, *Anaerococcus*, *Escherichia*, *Enterococcus*, *Bacteroides*, *Corynebacterium*, *Finegoldia*, *Pseudomonas*, *Peptoniphilus*, *or Sphingomonas* species were not statistically different between the two groups.

### 3.2. The Association of Bacterial Species in Uterine Endometrium Microbiome with Endometriosis in RIF

Multivariable logistic regression analysis was performed to determine whether the presence of *Dialister* or *Streptococcus* species in uterine endometrium microbiome was associated with endometriosis in RIF (Table 3). The results revealed that only *Dialister* species were found to be associated with endometriosis in RIF (odds ratio 10.97 [95% confidence interval 1.17–249.37], *p* = 0.036).

In the EM group, the number of bacterial species (median [range], 10 vs. 5 [6–11, 3–7], *p* = 0.021) and Shannon diversity index (0.50 vs. 0.20 [0.19–1.39, 0.03–0.46], *p* = 0.026) were higher in the five women with *Dialister* species than in the seven without *Dialister* species. The relative dominance rate of *Lactobacillus* species or prevalence of each bacterial species was not different between the two groups.

## 4. Discussion

This cohort study, for the first time, demonstrated that the number of bacterial species and rates of presence of *Dialister* and *Streptococcus* species increased in the uterine endometrium microbiome of women with RIF complicated by endometriosis as compared with those without endometriosis. The presence of *Dialister* species in women with RIF complicated by endometriosis was related to increases in the number of bacterial species and in the Shannon diversity index in the uterine endometrium microbiome. These results suggest that the presence of *Dialister* and *Streptococcus* species, together with increases in the number of bacterial species and Shannon diversity index in the uterine endometrium microbiome, are pathologically associated with RIF in women with endometriosis. Compared with women without endometriosis, those with endometriosis had dysbiosis in the gut microbiome with increases in the abundance of harmful bacteria and decreases in beneficial bacteria, resulting in a loss of bacterial diversity [25]. A study using 16S rRNA sequencing reported that the diversity of the uterine endometrium microbiome was higher in women with endometriosis who had surgically confirmed endometriosis lesions than in women with endometriosis but no confirmed lesions (symptomatic control) [26].

In the female reproductive tract, bacteria are classified into five community groups (community state types [CSTs] I–V), distinguished by the abundance of *Lactobacillus* species and the diversity of anaerobes [27,28]. Chen et al. reported that women with endometriosis had more CST IV bacteria than women without endometriosis [10]. Similarly, in the present study, the number of bacterial species and rates of presence of CST IV, *Dialister,* and *Streptococcus* species increased in the uterine endometrium microbiome of women with RIF complicated by endometriosis. The microbiome is known to play an integral role in the immune system, creating the permissive environment necessary for successful implantation. A complex microenvironment, influenced by infection and inflammation, is established by cytokines involved in both endometrial receptivity and embryo development [29].

*Dialister* species are anaerobic Gram-negative bacteria belonging to CST IV, found primarily in the human oral cavity and intestine, and are associated with bacterial vaginosis and preterm delivery [30]. *Dialister* species are also identified in the placentas of women with periodontal disease, spreading hematogenously from the oral cavity, and increase the risk of preterm delivery [31,32]. The presence of *Dialister* species in the intestinal microbiome reflects the activity and degree of inflammation in autoimmune diseases such as spondyloarthritis [33]. *Dialister* species may act on various immune cells to promote the production of proinflammatory cytokines and chemokines.

On the other hand, in inflammatory skin diseases such as atopic dermatitis and rosacea, *Dialister* is reported to act in a protective manner, because it produces propionic acid, induces regulatory T-cells, and controls inflammation by suppressing the production of inflammatory cytokines such as IL-6 and TNF [34], suggesting that the presence of *Dialister* species indicates the intensity of inflammation, but whether it is a cause or a consequence remains unclear at this stage. The presence of *Dialister* species reflects the inflammatory state and signifies an intrauterine environment that is difficult to implant; therefore, it is considered important to make the intrauterine environment *Lactobacilus* species dominant.

*Streptococcus* species is a Gram-positive coccus, also classified as CST IV, and is involved in bacterial vaginosis, preterm delivery, and neonatal infections [35]. *Streptococcus* species have been detected in the uterine endometrium microbiome of women with endometriosis [36] and in ovarian endometrioma [37]. Certain *Streptococcus* species are known to cause endometritis [38,39]. Since intrauterine inflammation prevents implantation by interrupting the endometrial switching of proliferation to differentiation, which is essential for embryo acceptance [40,41], treatment for dysbiosis with inflammation in the endometrium is required prior to embryo transfer.

Pathological endometriotic lesions include several sites, such as ovarian endometriomas, adenomyosis, and pelvic endometriosis. The endometrial tissue in adenomyosis is inflamed [42]. The prevalence of *Lactobacillus* species decreases, and bacteria belonging to CST IV increase in the uterine endometrium microbiome of women with adenomyosis [10]. In the present study, all four women with adenomyosis had *Dialister* and/or *Streptococcus* species. Women with endometriosis and adenomyosis have a high risk of preterm delivery, which may be caused by these bacteria in the intrauterine environment [43].

In RIF women with endometriosis, treatments with antibiotics, probiotics and prebiotics, as well as surgery and hormonal therapy for endometriosis may more effectively restore uterine endometrium to the *Lactobaccilus*-dominant microbiome.

This prospective study has several limitations. Since the number of RIF women with endometriosis is clearly smaller than the number of non-endometriosis cases, it is difficult to conclude that the results obtained from this study alone are correct. Further studies are needed and should enroll a larger sample of RIF women with endometriosis. The relationship between *Dialister* and/or *Streptococcus* species in uterine endometrium microbiome and endometriosis in women with RIF needs to be clarified to determine whether it is a cause or a consequence. Basic researches including several biomedical and pharmaceutical approaches will elucidate these pathologic mechanisms [44,45,46]. The investigation of implantation and pregnancy rates after treatments for abnormal uterine endometrium microbiome is needed in future studies.

## 5. Conclusions

This study was the first to reveal that the number of bacterial species and rates of presence of *Dialister* and *Streptococcus* species increased in the uterine endometrium microbiome of women with RIF complicated by endometriosis. These changes seemed to worsen the inflammatory state of the intrauterine environment, causing implantation failure. A major limitation of this study is the small number of women with endometriosis; therefore, future studies with a larger sample size are needed in order to prove the results of this study.

As a treatment strategy for RIF-complicated endometriosis, analyses of the uterine endometrium microbiome using 16S rRNA sequencing and treatments for endometriosis with surgery and/or hormonal therapy and for abnormal endometrium microbiome with antibiotics, prebiotics, and probiotics can be performed.

## Figures and Tables

**Table 1:** Clinical characteristics in repeated implantation failure women with and without endometriosis.

Clinical Characteristics	Women with Endometriosis (n = 12)	Women without Endometriosis (n = 31)	*p*-Value
Age, years	37.5 (29–44)	40 (33–45)	0.186
Body mass index, kg/m^2^	21.0 (19.1–35.4)	22.8 (16.4–35.4)	0.757
Gravity	1.0 (0–10)	2.0 (0–6)	0.779
Parity	0 (0–2)	0 (0–2)	0.913
Number of previous miscarriages	0.5 (0–8)	0 (0–5)	0.906
Number of implantation failures	3 (2–5)	4 (2–9)	0.176
Ovarian endometrioma	7 (58.3%)	0	N.A.
Adenomyosis	4 (33.3%)	0	N.A.
Pelvic endometriosis	3 (25.0%)	0	N.A.

Data are expressed as median (range) or numbers (percentages). The endometriosis case numbers were counted repeatedly. Statistical analyses were performed by Mann–Whitney U test and Fisher’s exact test. Abbreviation: N.A.—not applicable.

**Table 2:** Chronic endometritis and uterine endometrium microbiome in repeated implantation failure women.

	Women with Endometriosis n = 12	Women without Endometriosis n = 31	*p*-Value
Chronic endometritis (Liu’s method)	8.3% (1/12)	12.9% (4/31)	1.000
Plasma cell count/10 mm^2^ with CD138 staining	0.60 (0–25.61)	0.69 (0–75.83)	0.633
Number of bacterial species	6.0 (3–11)	3.0 (1–16)	0.009
Shannon diversity index	0.19 (0.03–1.39)	0.08 (0–1.75)	0.129
Relative dominance rate of *Lactobacillus* species, %	86.0 (0–99.8)	98.9 (0–100)	0.106
*Lactobacillus*-dominant microbiome (>90%)	5 (41.7%)	21 (63.6%)	0.222
Presence of *Lactobacillus* species	10 (83.3%)	30 (96.8%)	0.184
Relative dominance rate of *Lactobacillus crispatus*, %	0 (0–98.2)	0.15 (0–99.9)	0.053
Relative dominance rate of *Lactobacillus gasseri*, %	0 (0–99.7)	0 (0–96.2)	0.169
Relative dominance rate of *Lactobacillus iners*, %	0 (0–99.2)	0 (0–98.6)	0.833
Relative dominance rate of *Lactobacillus jensenii*, %	0 (0–0)	0 (0–98.3)	0.079
Presence of *Ureaplasma* species	2 (16.7%)	1 (3.3%)	0.184
Presence of *Mycoplasma* species	0 (0%)	0 (0%)	N.A.
Presence of *Gardnerella* species	2 (16.7%)	8 (25.8%)	0.698
Presence of *Prevotella* species	6 (50.0%)	7 (22.6%)	0.160
Presence of *Streptococcus* species	7 (58.3%)	5 (16.1%)	0.017
Presence of *Atopobium* species	1 (8.3%)	5 (16.1%)	0.659
Presence of *Dialister* species	5 (41.7%)	1 (3.3%)	0.004
Presence of *Bifidobacterium* species	3 (25.0%)	6 (19.4%)	0.692
Presence of *Anaerococcus* species	2 (16.7%)	2 (6.5%)	0.308
Presence of *Escherichia* species	0 (0%)	1 (3.3%)	N.A.
Presence of *Enterococcus* species	0 (0%)	1 (3.3%)	N.A.
Presence of *Bacteroides*	1 (8.3%)	1 (3.3%)	0.485
Presence of *Corynebacterium*	1 (8.3%)	5 (16.1%)	0.659
Presence of *Finegoldia*	4 (33.3%)	4 (12.9%)	0.185
Presence of *Pseudomonas*	0 (0%)	3 (9.7%)	0.548
Presence of *Peptoniphilus*	3 (25.0%)	2 (6.5%)	0.123
Presence of *Sphingomonas*	1 (8.3%)	3 (9.7%)	1.000

Data are expressed as median (range) or numbers (percentages). Statistical analyses were performed by Mann–Whitney U test and Fisher’s exact test. Abbreviation: N.A.—not applicable.

**Table 3:** Multivariable logistic regression analyses for bacteria species associated with endometriosis.

	Odds Ratio (95% Confidence Interval)	*p*-Value
*Dialister* species	10.97 (1.17–249.37)	0.036
*Streptococcus* species	3.59 (0.59–20.33)	0.155

Odds ratios were obtained using multivariable logistic regression analyses.

## Data Availability

The data underlying this study cannot be shared publicly for privacy reasons. The datasets generated and/or analyzed during the current study are available from the corresponding author on reasonable request.

## References

[1] Giudice L.C., Kao L.C. (2004). Endometriosis. Lancet.

[2] Macer M.L., Taylor H.S. (2012). Endometriosis and infertility: A review of the pathogenesis and treatment of endometriosis-associated infertility. Obstet. Gynecol. Clin. N. Am..

[3] Stephansson O., Kieler H., Granath F., Falconer H. (2009). Endometriosis, assisted reproduction technology, and risk of adverse pregnancy outcome. Hum. Reprod..

[4] Marchandot B., Curtiaud A., Matsushita K., Trimaille A., Host A., Faller E., Garbi O., Akladios C., Jesel L., Morel O. (2022). Endometriosis and cardiovascular disease. Eur. Heart J. Open.

[5] Missmer S.A., Cramer D.W. (2003). The epidemiology of endometriosis. Obstet. Gynecol. Clin. N. Am..

[6] Uzuner C., Mak J., El-Assaad F., Condous G. (2023). The bidirectional relationship between endometriosis and microbiome. Front. Endocrinol..

[7] Xu H., Liu M., Cao J., Li X., Fan D., Xia Y., Lu X., Li J., Ju D., Zhao H. (2019). The Dynamic Interplay between the Gut Microbiota and Autoimmune Diseases. J. Immunol. Res..

[8] Human Microbiome Project Consortium (2012). Structure, function and diversity of the healthy human microbiome. Nature.

[9] Vitale S.G., Ferrari F., Ciebiera M., Zgliczyńska M., Rapisarda A.M.C., Vecchio G.M., Pino A., Angelico G., Knafel A., Riemma G. (2022). The role of genital tract microbiome in fertility: A systematic review. Int. J. Mol. Sci..

[10] Chen C., Song X., Wei W., Zhong H., Dai J., Lan Z., Li F., Yu X., Feng Q., Wang Z. (2017). The microbiota continuum along the female reproductive tract and its relation to uterine-related diseases. Nat. Commun..

[11] Franasiak J.M., Werner M.D., Juneau C.R., Landis J., Zhan Y., Treff N.R., Scott R.T. (2016). Endometrial microbiome at the time of embryo transfer: Next-generation sequencing of the 16S ribosomal subunit. J. Assist. Reprod. Genet..

[12] Moreno I., Codoñer F.M., Vilella F., Valbuena D., Martinez-Blanch J.F., Jimenez-Almazán J., Alonso R., Alamá P., Remohí J., Pellicer A. (2016). Evidence that the endometrial microbiota has an effect on implantation success or failure. Am. J. Obstet. Gynecol..

[13] Liu Y., Ko E.Y., Wong K.K., Chen X., Cheung W.C., Law T.S., Chung J.P., Tsui S.K., Li T.C., Chim S.S. (2019). Endometrial microbiota in infertile women with and without chronic endometritis as diagnosed using a quantitative and reference range-based method. Fertil. Steril..

[14] Lozano F.M., Lledo B., Morales R., Cascales A., Hortal M., Bernabeu A., Bernabeu R. (2023). Characterization of the Endometrial Microbiome in Patients with Recurrent Implantation Failure. Microorganisms.

[15] Gao X., Louwers Y., Laven J.S.E., Schoenmakers S. (2024). Clinical Relevance of Vaginal and Endometrial Microbiome Investigation in Women with Repeated Implantation Failure and Recurrent Pregnancy Loss. Int. J. Mol. Sci..

[16] Toson B., Simon C., Moreno I. (2022). The Endometrial Microbiome and Its Impact on Human Conception. Int. J. Mol. Sci..

[17] Takimoto K., Yamada H., Shimada S., Fukushi Y., Wada S. (2023). Chronic Endometritis and Uterine Endometrium Microbiota in Recurrent Implantation Failure and Recurrent Pregnancy Loss. Biomedicines.

[18] Lee S.R., Lee J.C., Kim S.H., Oh Y.S., Chae H.D., Seo H., Kang C.S., Shin T.S. (2021). Altered Composition of Microbiota in Women with Ovarian Endometrioma: Microbiome Analyses of Extracellular Vesicles in the Peritoneal Fluid. Int. J. Mol. Sci..

[19] Leonardi M., Hicks C., El-Assaad F., El-Omar E., Condous G. (2020). Endometriosis and the Microbiome: A Systematic Review. BJOG Int. J. Obstet. Gynaecol..

[20] Ma J., Gao W., Li D. (2023). Recurrent implantation failure: A comprehensive summary from etiology to treatment. Front. Endocrinol..

[21] Shi Y., Tanimura K., Sasagawa Y., Yamada H. (2020). Vaginal microbiota associated with preterm delivery. J. Infect. Chemother..

[22] Kyono K., Hashimoto T., Nagai Y., Sakuraba Y. (2018). Analysis of endometrial microbiota by 16S ribosomal RNA gene sequencing among infertile patients: A single-center pilot study. Reprod. Med. Biol..

[23] Kim B.R., Shin J., Guevarra R.B., Lee J.H., Kim D.W., Seol K.H., Lee J.H., Kim H.B., Isaacson R.E. (2017). Deciphering diversity indices for a better understanding of microbial communities. J. Microbiol. Biotechnol..

[24] Schloss P.D., Handelsman J. (2006). Introducing SONS, a tool for operational taxonomic unit-based comparisons of microbial community memberships and structures. Appl. Environ. Microbiol..

[25] Ser H.L., Yong S.J.A., Shafiee M.N., Mokhtar N.M., Ali R.A.R. (2023). Current Updates on the Role of Microbiome in Endometriosis: A Narrative Review. Microorganism.

[26] Wessels J.M., Domínguez M.A., Leyland N.A., Agarwal S.K., Foster W.G. (2021). Endometrial microbiota is more diverse in people with endometriosis than symptomatic controls. Sci. Rep..

[27] Smith S.B., Ravel J. (2017). The vaginal microbiota, host defence and reproductive physiology. J. Physiol..

[28] Madere F.S., Sohn M., Winbush A.K., Barr B., Grier A., Palumbo C., Java J., Meiring T., Williamson A., Bekker L.G. (2022). Transkingdom Analysis of the Female Reproductive Tract Reveals Bacteriophages form Communities. Viruses.

[29] Robertson S.A., Chin P.Y., Glynn D.J., Thompson J.G. (2011). Peri-conceptual cytokines—Setting the trajectory for embryo implantation, pregnancy and beyond. Am. J. Reprod. Immunol..

[30] Ravel J., Gajer P., Abdo Z., Schneider G.M., Koenig S.S.K., McCulle S.L., Karlebach S., Gorle R., Russell J., Tacket C.O. (2011). Vaginal microbiome of reproductive-age women. Proc. Natl. Acad. Sci. USA.

[31] Offenbacher S., Katz V., Fertik G., Collins J., Boyd D., Maynor G., McKaig R., Beck J. (1996). Periodontal infection as a possible risk factor for preterm low birth weight. J. Periodontol..

[32] Barak S., Oettinger-Barak O., Machtei E.E., Sprecher H., Ohel G. (2007). Evidence of periopathogenic microorganisms in placentas of women with preeclampsia. J. Periodontol..

[33] Tito R.Y., Cypers H., Joossens M., Varkas G., Van Praet L., Glorieus E., Van den Bosch F., De Vos M., Raes J., Elewaut D. (2017). Dialister as a Microbial Marker of Disease Activity in Spondyloarthritis. Arthritis Rheumatol..

[34] Zhong Y., Wang F., Meng X., Zhou L. (2024). The associations between gut microbiota and inflammatory skin diseases: A bi-directional two-sample Mendelian randomization study. Front. Immunol..

[35] Bayar E., Bennett P.R., Chan D., Sykes L., MacIntyre D. (2020). The pregnancy microbiome and preterm birth. Semin. Immunopathol..

[36] Khan K.N., Fujishita A., Kitajima M., Hiraki K., Nakashima M., Masuzaki H. (2014). Intra-uterine microbial colonization and occurrence of endometritis in women with endometriosis. Hum. Reprod..

[37] Khan K.N., Fujishita A., Masumoto H., Muto H., Kitajima M., Masuzaki H. (2016). Molecular detection of intrauterine microbial colonization in women with endometriosis. Eur. J. Obstet. Gynecol. Reprod. Biol..

[38] Weckel A., Guilbert T., Lambert C., Plainvert C., Goffinet F., Poyart C., Méhats C., Fouet A. (2021). Streptococcus pyogenes Infects Human Endometrium by Limiting the Innate Immune Response. J. Clin. Investig..

[39] Memish Z.A., Gravel-Tropper D., Oxley C., Toye B., Garber G.E. (1994). Group A Streptococcal Endometritis: Report of an Outbreak and Review of the Literature. Can. J. Infect. Dis..

[40] Ono Y., Yoshino O., Hiraoka T., Sato E., Fukui Y., Ushijima A., Nawaz A., Hirota Y., Wada S., Tobe K. (2020). CD206+ M2-Like Macrophages Are Essential for Successful Implantation. Front. Immunol..

[41] Hirota Y. (2019). Progesterone Governs Endometrial Proliferation-Differentiation Switching and Blastocyst Implantation. Endocr. J..

[42] Kobayashi H. (2023). Endometrial Inflammation and Impaired Spontaneous Decidualization: Insights into the Pathogenesis of Adenomyosis. Int. J. Environ. Res. Public Health.

[43] Horton J., Sterrenburg M., Lane S., Maheshwari A., Li T.C., Cheong Y. (2019). Reproductive, obstetric, and perinatal outcomes of women with adenomyosis and endometriosis: A systematic review and meta-analysis. Human Reprod. Update.

[44] Tong F., Zhou Y., Xu Y., Chen Y., Yudintceva N., Shevtsov M., Gao H. (2023). Supramolecular Nanomedicines Based on Host-Guest Interactions of Cyclodextrins. Exploration.

[45] Wu S., Yan M., Liang M., Yang W., Chen J., Zhou J. (2023). Supramolecular Host-Guest Nanosystems for Overcoming Cancer Drug Resistance. Cancer Drug Resist..

[46] Liu L., Xiong M., Rong Q., Zhang M., Zhang X.B. (2023). Nucleic Acid Sensors in Vivo: Challenges and Opportunities. View.

